# Adjustment of speaker’s referential expressions to an addressee’s likely knowledge and link with theory of mind abilities

**DOI:** 10.3389/fpsyg.2015.00823

**Published:** 2015-06-17

**Authors:** Amélie M. Achim, Marion Fossard, Sophie Couture, André Achim

**Affiliations:** ^1^Département de Psychiatrie et de Neurosciences, Université Laval, Québec, QCCanada; ^2^Centre de Recherche de l’Institut Universitaire en Santé Mentale de Québec, Québec, QCCanada; ^3^Institut des Sciences du Langage et de la Communication, Université de Neuchâtel, NeuchâtelSwitzerland; ^4^École de Psychologie, Université Laval, Québec, QCCanada; ^5^Département de Psychologie, Université du Québec à Montréal, Montreal, QCCanada

**Keywords:** reference, theory of mind, mentalizing, interactive task, collaboration, common ground, egocentric bias

## Abstract

To communicate cooperatively, speakers must determine what constitutes the common ground with their addressee and adapt their referential choices accordingly. Assessing another person’s knowledge requires a social cognition ability termed theory of mind (ToM). This study relies on a novel referential communication task requiring probabilistic inferences of the knowledge already held by an addressee prior to the study. Forty participants were asked to present 10 movie characters and the addressee, who had the same characters in a random order, was asked to place them in order. ToM and other aspects of social cognition were also assessed. Participants used more information when presenting likely unknown than likely known movie characters. They particularly increased their use of physical descriptors, which most often accompanied movie-related information. Interestingly, a significant relationship emerged between our ToM test and the increased amount of information given for the likely unknown characters. These results suggest that speakers use ToM to infer their addressee’s likely knowledge and accordingly adapt their referential expressions.

## Introduction

Models of language production suggest that to communicate cooperatively, speakers must determine what constitutes the common ground with their addressee and adapt their referential choices accordingly (Clark et al., 1983). Common ground can be defined as the information (e.g., knowledge or beliefs) that is shared between a speaker and his addressee. Common ground information can be shared because of community membership or based on prior common experiences such as seeing or hearing something together or having previously interacted on the same topic (Clark et al., 1983). During the course of a conversation, a collaborative speaker can take common ground into account to adjust the formulation of his references, i.e., formulate references that will be understood by the addressee yet avoid overly specific references that take longer to produce and would violate Grice’s principle of quantity (Grice, 1975).

Even though this adjustment is not perfect, with speakers sometimes using privileged information (e.g., Horton and Keysar, 1996; Wu and Keysar, 2007), several studies have shown that people do adapt their referential expressions to their addressee. For instance, there is evidence that speakers adjust their messages when speaking to someone who shows a good knowledge on a given topic (Isaacs and Clark, 1987), when speaking to someone with a foreign accent (Kingsburry, 1968, cited in Krauss, 1987), when speaking to close friends (Fussell and Krauss, 1985, cited in Krauss, 1987), when speaking to a child (Brand et al., 2002), or when speaking to someone with previously established shared knowledge (Galati and Brennan, 2010; Heller et al., 2012; Gorman et al., 2013).

In their seminal study, Isaacs and Clark (1987) gave pairs of participants two identical sets of postcards of common New York scenes. One participant acted as the director who had to describe the scenes in a predetermined order so that the other participant, the matcher, could arrange his cards in the same order. Some matchers had a good knowledge of New York while other matchers did not. The study showed that the directors quickly adapted how they presented the postcards depending on their matcher’s knowledge, using their partner’s responses to adjust quickly after the beginning of the task. More specifically, the directors used more proper names of places when interacting with expert interlocutors and more physical descriptions of places with novice interlocutors.

A more recent study by Heller et al. (2012) also showed that people adapt the content of their referential expressions based on the information that is shared or not with their addressee. In that study, the speakers were asked to help their addressee identify a series of target shapes among sets of three abstract shapes. Common ground was manipulated by having pairs of participants learn together the names of a certain number of abstract shapes before the task while the speaker learned other shape names alone. Speakers used more names for the shapes for which the other participant had also learnt the names. Interestingly, names were also used for shapes for which only the participant had learnt the name, but shape names were in this case typically accompanied by descriptive information, suggesting that participants were able to distinguish shared from privileged information. Another study by Gorman et al. (2013) also used an analogous design and observed similar results.

These studies together suggest that speakers do adapt their referential expressions depending on the information that is part of the common ground with their addressee, a phenomenon sometimes designated as audience design or recipient design (e.g., Horton and Gerrig, 2002; Newman-Norlund et al., 2009; Brennan et al., 2010). Audience design has also been reported for other aspects of communicative behavior, including for instance adjustments of prosody or intonation, adjustments in the amount of information provided or in the repetition of the same information (Grieser and Kuhl, 1988; Brand et al., 2002; Newman-Norlund et al., 2009). As Heller et al. (2012) observed, if the verbal production choices made by a speaker depend on common ground, then during normal everyday conversations speakers must determine what information is in common ground with their addressees (i.e., shared information) and what information is not (i.e., privileged information). Assessing another person’s mental states, including their state of knowledge or their beliefs, depends on a social cognition ability called theory of mind (ToM; also known as mentalizing; Achim et al., 2013a; Pinkham et al., 2014).

Theory of mind has garnered a lot of attention in developmental studies, which showed that by the age of four most children have acquired the ability to assess other people’s beliefs and distinguish them from their own (Bruneau-Bhérer et al., 2012). Even once this ability has been acquired, inter-individual differences can be observed in how good adults are at identifying the mental states of others in given situations (ex: Achim et al., 2012, 2013a; Pinkham et al., 2014). In typical ToM tasks intended for adult populations, participants are presented with short stories and are then asked to answer questions about the characters’ likely mental states, including their beliefs, knowledge, intentions or emotions, in different situations. Such ToM tasks typically rely on non-interactive materials, however, and participants have to assess the mental states of fictitious characters with whom they will never truly interact. In a recent systematic review (Achim et al., 2013a), we highlighted that very few studies have assessed ToM during participative interactions and so we know little of how these abilities are deployed during real social exchanges.

While the evidence described above shows that speakers adapt their referential choices based on the addressee’s knowledge, we are aware of only one study that has directly examined the relationship between ToM abilities and referential choices during a realistic verbal interaction. In that study, Champagne-Lavau et al. (2009) used a referential communication paradigm, which reproduces a communication situation that implies a social interaction based on the collaboration between two partners. Their version of the referential communication paradigm triggered a conversation around two sets of tangrams (abstract shapes) that were each discussed on four consecutive occasions. That study not only confirmed that healthy people adapt their referential expressions depending on whether the referent has previously been introduced or not to their addressee (first versus subsequent trials), but also that people with ToM deficits, in that case a group of patients with schizophrenia, do not adapt their expressions to the same extent. Moreover, this reduced adjustment was particularly prominent in patients with more important deficits on a standard ToM task. However, in that study it is possible that speakers employed more indefinite markers on the first trial and more definite markers on the subsequent trial because of their own increased familiarity with the tangram shapes as the task progressed. Moreover, the ToM test used for that study did not allow inter-individual differences in the healthy participant’s group to be identified as the ToM task used in that study was relatively easy and is known to present ceiling effects in healthy individuals. Overall, Champagne-Lavau’s study nonetheless suggested links between ToM abilities and the adjustment of referential choices, though this relationship should minimally be confirmed, ideally while controlling for speakers’ own knowledge and using a referential communication task that places greater emphasis on the assessment of addressees’ knowledge.

The present study thus had two main objectives. First, we aimed to examine whether speakers adapt the content of their referential expressions when the addressee’s knowledge has not been previously established and has to be reassessed on an item-by-item basis for each referent introduced during the task. As Galati and Brennan (2010, p. 47) have previously observed, the previous studies that provided evidence for audience design have typically relied on tasks in which speakers could “*represent relevant aspects of common ground in a simple, clear way.*” For example, previous studies either held the addressee’s knowledge constant across all items of the task (e.g., the addressee had an overall good or poor knowledge of New York scenes in the study by Isaacs and Clark, 1987) or clearly established the other person’s knowledge before the task (as in the study by Heller et al., 2012 where participants learned together the names of a certain number of abstract shapes). It thus remains unclear whether a spontaneous adjustment to the addressee’s needs would also be observed for referents that require constant reassessment of the addressee’s *likely* knowledge, i.e., for probabilistic evaluations of common ground. Determining if an adjustment also occurs in such circumstances is important as during real life conversations, the referents being mentioned are not restricted to a specific stimulus category and can vary widely in terms of the likelihood that a given addressee will know each referent. In this study, the referents were movie characters that were discussed for the first time between the speaker and addressee, and these characters varied in terms of how likely it was that the addressee would know them. Brown-Schmidt (2012) recently suggested that common ground can be represented in a graded, probabilistic way. Though the evidence that she presented to support her view were derived from an eye-tracking study during reference interpretation, a probabilistic view of common ground could certainly also apply to language production. We thus hypothesized that an adjustment “for-the-addressee” (Galati and Brennan, 2010) would be observed for each item of our task such that speakers would use more movie related information when their addressee is more likely to know the referent than when he is less likely to know the referent. We further expected that the movie-related information used for likely unknown-referents would more often be accompanied by additional descriptive information, similarly to the pattern observed by Heller et al. (2012). In order to distinguish adjustments that occur “for-the-addressee” and adjustments that occur “for-the-speaker” (i.e., because they are easier for the speaker to produce), we controlled for participants’ knowledge when assessing the effect of the addressee’s likely knowledge. Moreover, we also directly examined adjustments related to the participants’ own familiarity with the movies, expecting that participants would use mainly descriptive information to present movie characters that they themselves did not know but then use a wider range of information to present characters that they did know.

Given the suggested relationship between choices of referential expressions and ToM (e.g., Brennan et al., 2010), the second objective of this study was to assess the relationship between the adjustments of referential choices based on an addressee’s likely knowledge and ToM abilities in healthy individuals using a more sensitive ToM measure than the one employed by Champagne-Lavau et al. (2009). Other aspects of social cognition were also examined in our study including social knowledge, emotion recognition, and empathic perspective-taking. We expected a relationship between adjustment of verbal productions to the addressee’s likely knowledge and both ToM and empathic perspective-taking. In contrast, we did not predict a strong relationship with our measures of social knowledge or emotion recognition as these tests assess lower-level aspects of social cognition that are less directly related to social functioning (Achim et al., 2012, 2013b).

## Materials and Methods

### Participants

Forty participants took part in this study. All were native French speakers aged between 18 and 40 years old (mean age = 24.2; 32 men; mean education = 14.2 years). Participants were not included in the study if they reported a history of neurological disorder, head trauma or psychiatric disorder or if taking a psychoactive medication. The local ethics board approved the study and all participants signed an informed consent.

### Procedure

Because our experimental task is based on movies (see below), participants first completed a movie knowledge questionnaire, then completed a social cognition assessment (as well as additional tests and questionnaires not reported here) and finally performed our experimental task. Our assessments are presented in more details below.

### Experimental Task

**FIGURE 1:**
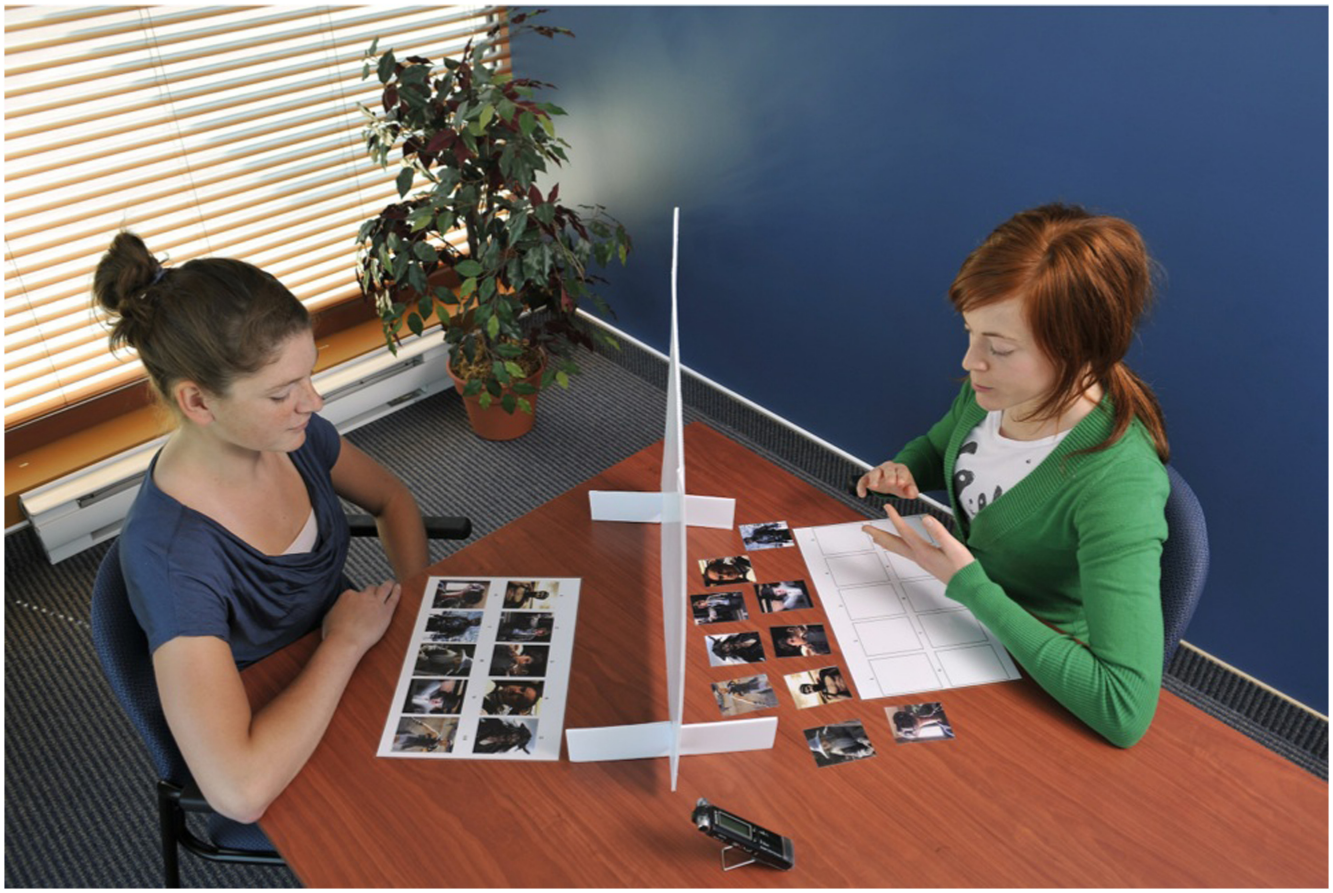
**Illustration of the procedure used for this study.** The participant (on the left) has 10 images placed in a predetermined order. The addressee (on the right) has the same images in a random order and has to replace them in the correct order based on the verbal utterances of the participants.

The current project is based on the referential communication paradigm (e.g., Clark et al., 1983; Champagne-Lavau et al., 2009). An opaque screen is placed between the two partners to prevent non-verbal communication during the verbal interaction and both partners are presented with an identical set of image cards. For this study, participants received 10 image cards in a predetermined order whereas their addressee received 10 cards with the same images in a random order, as illustrated in **Figure 1**. Participants had to present each card in the given order so that their addressee could replace his set in the same order. The addressee could give some feedback to signal understanding (e.g., “*ok*”) or to point out misunderstandings or ambiguities (e.g., “*can you give me more details*”), and the participant could provide additional information.

The addressee was always a woman in her twenties, and the material consisted of 10 images of movie characters, all male (see **Table 1**), taken from movies that can either safely be assumed to be known by a woman in her twenties (the likely known condition, five images) or not safely assumed to be known by a woman in her twenties (the likely unknown condition, five images). Classification of the movies into the likely known or likely unknown categories was confirmed by two surveys. The first survey included 27 movies titles. Thirty-three participants (aged between 20 and 30 years old, 14 men) indicated if they had seen the movie or not and whether they would assume that most women in their 20s had seen the movie. This allowed us to eliminate the movies for which there was little agreement on whether it was likely known or not (nine movies) and those that had been seen by too few people (six movies). For the remaining 12 movies, we performed a second survey in which 54 participants (aged between 18 and 30 years old, 32 men) were again questioned about the movies but also about the main male characters in each movie, confirming that the movies and their main characters were correctly classified into the likely known (five movies) and likely unknown (seven movies) categories. From these 12 movies, 10 were selected for our final stimulus set (five likely known and five likely unknown).

**Table 1:** List of characters that the participants had to present.

Character	Movie
**Likely-known condition**
Harry Potter	Harry Potter and the Philosopher’s Stone
Jack Sparrow	Pirates of the Caribbean
E.T.	E.T. The Extra-Terrestrial
Gandalf	The Lord of the Rings
Maximus	Gladiator
**Likely-unknown condition**
Leonidas	300
Martin Riggs	Lethal Weapon
Don Vito Corleone	The Godfather
Wolverine	X-men
Alex	A Clockwork Orange

The role of the addressee was here held by a trained research assistant to standardize the feedback given for the different items in the card set (see below). To avoid that the participants assume all image cards to be known from the assistant-addressee (i.e., the assistant could learn the name of a likely unknown characters when administering the task), a concealment strategy was developed to make the participants believe that their addressee did the task with different images for each participant (when in fact the same 10 images were always used). More specifically, the images were presented in a sealed envelope allegedly prepared by another research assistant and participants were told that their addressee had previously done the task but each time with a different set of images, so they had not been exposed to the images that were in the envelop in the context of the study. A pilot study previously confirmed the success of this strategy, with none of the 10 pilot subjects reporting suspecting that the addressee was familiar with the material used for the task.

During the task, participants were asked to present the characters to the addressee one at a time. The addressee knew all the characters in the likely known condition and acted as if she did not know the characters in the likely unknown condition. More specifically, she was trained to ignore any movie-related information provided for the characters in the likely unknown condition (ex: character’s name or role in the movie, or movie title) regardless of her own knowledge. She was allowed to give verbal feedback, for example saying “ok” or asking for additional information, but was asked to never mention any information about the characters. This material and procedure was created so that participants had to take into consideration the knowledge that they can attribute to the addressee for each of the different movie characters (i.e., which movie character’s are likely known by their addressee) on an image-by-image basis in order to produce appropriate referential information. Participants were requested not to name the actors, and to really focus on the characters.

### Movie Knowledge Questionnaire

A self-report movie questionnaire was administered to determine, for each movie from which we had extracted our stimuli, whether the participant had seen it or not. Participants who reported seeing the movie were also asked how long ago they had seen it and to estimate the percentage of women between 20 and 30 years old in Quebec they thought had also seen the movie. This movie knowledge questionnaire was always done first followed by the social cognition assessment, the other measures not reported here and then the experimental task. This order was chosen because doing the questionnaire after the task may have led the participants to answer based on their experience with the addressee during the task, and also because we wanted to introduce a delay between the questionnaire and the experimental task (at least 90 min to complete the full set of other measures).

### Social Cognition Assessment

Our social cognition assessment included measures of ToM, social knowledge and emotion recognition, measured with the tests from the Batterie Intégrée de Cognition Sociale (BICS; Achim et al., 2012) and empathic perspective-taking measured with the Interpersonal Reactivity Index (IRI; Davis, 1980).

#### Theory of Mind Assessment

The combined stories test from the BICS (Achim et al., 2012) is a validated ToM measure that includes a combination of stories that cover attributions of a full range of mental states, including beliefs/knowledge, intentions/desires, and emotions. Each item consists of a short story depicting at least two characters involved in a specific situation. For each item, participants are asked to read the story aloud and are then asked one or two open ToM questions per story. Answers to these questions require making inferences about the characters’ mental states (e.g., their intentions, beliefs, knowledge, or emotions), and answers are scored 2, 1, or 0 points according to a validated correction grid. A score of 2 is given for a complete and explicit answer that takes the mental states of the characters into account, a score of 1 reflects an incomplete answer, and a score of 0 an incorrect answer. This test was selected because it includes a large number of items and does not suffer from a ceiling effect in healthy participants (Achim et al., 2012, 2013b; Lavoie et al., 2014).

#### Social Knowledge Assessment

Social knowledge was assessed by presenting 14 hypothetical situations and asking participants how people in general would react in these situations. The situations include for example “Someone who learns that he has been lied to.” Answers were scored 0 or 1 point according to a validated correction grid (Achim et al., 2012).

#### Emotion Recognition Assessment

Emotion recognition was assessed by presenting 14 emotional facial expressions (Ekman and Friesen, 1976) and asking participants to identify, from seven possible labels, which emotion is being expressed. One point was given for a correct answer and 0 for incorrect answers.

#### Perspective-Taking

Perspective-taking was assessed with the IRI (Davis, 1980), a widely used self-report questionnaire that includes four scales that measure four aspects of empathy: (1) Perspective taking; (2) Fantasy; (3) Empathic concern; and (4) Personal distress. The Perspective taking scale was of particular interest here. This scale includes seven items for which participants have to determine to what extent each statement describes them and provide a rating on a five-point scale (from 0 – does not describe me well to 4 – describes me very well). It includes items such as “Before criticizing somebody, I try to imagine how I would feel if I were in their place.” Validity of the IRI has been well documented (Davis, 1983).

### Data Processing and Analyses

For the experimental task, the interactions were tape-recorded and then transcribed verbatim. For each of the 10 images, we coded each piece of information provided to present each character according to whether it was (a) the character’s name (e.g., “Jack Sparrow”); (b) the title of the movie with that character (e.g., “Pirates of the Caribbean”); (c) the role of the character in the movie (e.g., “a Pirate”); (d) a physical descriptor (e.g., “has a red shirt”). It was often the case that multiple pieces of information were provided for the same character (e.g., it’s a pirate with a red shirt, his name is Jack Sparrow”) and all pieces of information were taken into account in our coding, excluding, however, the information added after a clarification demand from the addressee (i.e., only the spontaneous presentations made for each character were subjected to our analyses) since we wanted to focus on the utterances produced in advance of explicit verbal feedback about the communicative success or failure.

The variables retained for analyses included: (1) the number of pieces of information provided per character; (2) the proportion of trials that included each of the four types of information content, including physical descriptors as well as each of the three types of movie-related information (i.e., names, movie titles and roles); and, given some trials included more than one type of information, (3) the context in which descriptors or movie-related information were used including the proportion of cases where participants employed physical descriptors only (descriptors without movie information), movie information only (without physical descriptors, but including some trials with more than one type of movie information, for example name and movie title) or descriptors in combination with movie related information. Given the limited number of trials in this study, we chose to combine together in that third category trials in which the movie information was followed by descriptors and trials in which description was followed by movie related information.

Even if we were primarily interested in the adjustment for the addressee, we recognize that an adjustment is also likely to occur for the participant himself (i.e., because some information is easier for the participants to produce) and we thus aimed to assess both the effect of the likely known or likely unknown nature of the stimuli for the addressee and the effect of the participant’s personal knowledge of the movie characters.

Participants had seen a vast majority of the likely known movies, leaving too few unseen likely known movies for an eventual 2 × 2 analysis of variance that would incorporate personal knowledge and likely knowledge of the addressee in a single analysis. For this reason, our strategy to distinguish the effect of the participant’s personal knowledge and the effect of the addressee’s likely knowledge was the following.

For each of our variables, *t*-test were used to (1) compare movies that participants had seen versus not seen, performed only on the likely unknown trials to control for the addressee’s likely knowledge and (2) compare likely known versus likely unknown characters, performed only for the movies that the participant had seen in order to control for their personal knowledge. This strategy was crucial to be able to distinguish the effect of the participant’s personal knowledge from the effect of their evaluation of the addressee’s likely knowledge. However, for the comparison of likely known versus likely unknown it led to the exclusion of five participants who had seen none of the likely unknown movies. For the comparison of seen versus not seen movies, it also led to the exclusion of the same five participants who had seen no likely unknown movies plus four others who had seen all the likely unknown movies.

Another important consideration is that because of the small number of trials involved, the data were not satisfactorily distributed for a reliable parametric assessment of the null hypothesis for our paired *t*-tests. The probability of the observed *t*-value under the hypothesis that participant’s personal knowledge or addressee’s likely knowledge did not matter was thus assessed from the rank of its absolute value of *t* among absolute values of *t* obtained for random reassignment of signs to the observed differences. Typically, 1000 random sign reassignments were performed. If, however, the observed rank was then within the 95% confidence interval of the set significance threshold (alpha = 0.05), the number of sign reassignments was raised to 20000 to get a finer evaluation of the probability.

In a second step, we also assessed the correlations between (1) the degree of adjustment computed for each subject and each variable by contrasting the two trial categories (i.e., score on the variable for likely known characters minus score for likely unknown characters; or score on the variable for seen movies minus score for unseen movies), and (2) the scores on our four social cognition measures, namely the ToM performance scores from the Combined Stories task, the Perspective-Taking scores from the IRI, the social knowledge performance scores, and the emotion recognition performance scores. Here again, we used permutations to better estimate the probability of the observed correlations. If social cognition abilities such as ToM indeed contribute to the assessment of common ground and hence to reference adjustments, participants with better social cognition performance should show more pronounced adjustment as a function of the addressee’s likely knowledge, and be less influenced by their own knowledge.

## Results

### Results from the Movie Questionnaire

Participants had seen between 1 and 10 movies, with a mean of 6.7 movies (SD = 2.3) per participant (only 15% of our participants had seen less than half of the movies). When considering separately our likely known and likely unknown movies, participants had seen a mean of 4.3 likely known movies (SD = 1.2) and 2.6 likely unknown movies (SD = 1.6).

When asked to determine the proportion of women in their twenties having seen the movies (asked only for movies the participants had themselves seen), the expected pattern of responses was observed, with the movies previously classified as likely known more often considered as having been seen by a majority of women in their twenties (65.6%) than the movies previously classified as likely unknown (16.1%).

### Results for the Number of Pieces of Information

As presented in **Table 2**, the number of pieces of information included when presenting the characters was not significantly affected by participants’ own knowledge but was significantly influenced by the addressees’ likely knowledge.

**Table 2:** Effect of the participant’s personal knowledge (seen or not seen) and of the addressee’s likely knowledge (likely known or likely unknown) on the number of pieces of information.

Number of pieces of information per character		Paired *t*-test	Correlation ToM^1^	Correlation IRI-PT^1^
**Seen**	**Not seen**			
2.27	2.55	*t*(30) = 1.37, N.S.	*r* = 0.23, N.S.	*r* = 0.02, N.S.
**Likely known**	**Likely unknown**			
1.79	2.29	*t*(34) = 3.45, *p* < 0.001	*r* = −0.47, *p* < 0.003	*r* = −0.29, N.S.
				

*ToM, Theory of Mind. IRI-PT, the empathic perspective-taking scale from the Interpersonal Reactivity Index (Davis, 1980).*

*^1^A positive correlation means that participants with a higher score on the social cognition measures used relatively more information for the Seen or for the Likely known conditions, while a negative correlation reflects that they used relatively more information for the Not seen or the Likely unknown condition.*

Interestingly, this adjustment to the addressees’ likely knowledge was significantly correlated to performances on the ToM test, such that participants with better ToM abilities showed a greater increase in the amount of information provided for the likely unknown characters relative to the likely known characters. This correlation was strong enough to survive a Bonferroni correction for multiple testing (see **Table 2**). No other significant correlations were observed between our other social cognition measures and the number of pieces of information, but the correlations with the Perspective taking scale are nonetheless included in **Table 2** given our a priori hypotheses for this measure.

### Results for the Four Types of Information Content

**Table 3** shows that when the participants had not seen the movie they most often used physical descriptors whereas for movies they had seen they most often employed movie related information, in particular the characters’ names and movie titles. For the movies that the participants had seen, there was nonetheless an adjustment to the addressee’s likely knowledge. First, participants more often used physical descriptors for likely unknown characters. Second, though there was no overall difference in the use of movie related information between likely known and likely unknown characters (when considering together either names, roles, or movie titles), the type of movie related information being used was affected by the addressee’s likely knowledge. More precisely, names were more often used for likely known characters whereas movie titles were more often used for likely unknown characters (see **Table 3**).

**Table 3:** Effect of the participant’s personal knowledge (seen or not seen) and of the addressee’s likely knowledge (likely known or likely unknown) on the use of four types of information content.

Proportion of characters with use of:			Paired *t*-test^1^	Correlation ToM^2^	Correlation IRI-PT^2^
	**Seen**	**Not seen**			
Physical descriptors	0.51	0.79	*t*(30) = 3.83, *p* < 0.001	*r* = 0.06, N.S.	*r* = −0.15, N.S.
Any movie information	0.90	0.43	*t*(30) = 6.12, *p* < 0.001	*r* = -0.11, N.S.	*r* = -0.17, N.S.
Names	0.48	0.08	*t*(30) = 5.17, *p* < 0.001	*r* = 0.07, N.S.	*r* = 0.09, N.S.
Movie titles	0.50	0.29	*t*(30) = 2.79, *p* < 0.03	*r* = 0.10, N.S.	*r* = −0.00, N.S.
Roles	0.24	0.13	*t*(30) = 1.79, N.S.	*r* = 0.04, N.S.	*r* = −0.21, N.S.
	**Likely known**	**Likely unknown**			
Physical descriptors	0.36	0.55	*t*(34) = 3.43, *p* < 0.001	*r* = −0.14, N.S.	*r* = −0.23, N.S.
Any movie information	0.89	0.87	*t*(34) = 0.60, N.S.	*r* = −0.16, N.S.	*r* = 0.33, N.S.
Names	0.65	0.47	*t*(34) = 2.72, *p* < 0.016	*r* = 0.12, N.S.	*r* = −0.04, N.S.
Movie titles	0.27	0.45	*t*(34) = 2.64, *p* < 0.021	*r* = −0.24, N.S.	*r* = 0.06, N.S.
Roles	0.21	0.27	*t*(34) = 1.49, N.S.	*r* = −0.30, N.S.	*r* = 0.01, N.S.

*ToM, Theory of Mind. IRI-PT, the empathic perspective-taking scale from the Interpersonal Reactivity Index (Davis, 1980). ^1^Participants who did not have data in both categories for a given comparison were excluded from the analyses. ^2^A positive correlation means that participants with a higher score on the social cognition measures used relatively more of the targeted information type for the Seen or for the Likely known conditions, while a negative correlation reflects that they used relatively more of that information type for the Not seen or the Likely unknown condition.*

No significant correlation was observed between the adjustment in these measures and performance on the social cognition tests. Given our a priori hypotheses, the correlation results are nonetheless included in **Table 3** for ToM and Perspective taking.

These first analyses included all information provided without taking into account its place in the sequence. Additional analyses were thus conducted while considering only the first element of information mentioned for each trial, looking at the effect of the likely known versus likely unknown characters manipulation (e.g., only the name was considered if a character was presented by his name followed by a physical description). These analyses showed that participants more often started their presentation with physical descriptors for likely unknown trials (23%) than for the likely known trials (12%), although this effect did not reach significance [*t*(34) = 2.0, *p* < 0.055]. Similarly, participants more often started their presentation with names for the likely known characters (58%) than likely unknown characters (44%), although this effect was also only at a trend level [*t*(34) = 1.72, *p* < 0.067]. There was no evidence of an effect for movie titles (*p* = 0.80) or for roles (*p* = 0.45), and the effect for any movie information did not reach significance (*p* = 0.14).

### Results for the Combinations of Information

Results regarding the combinations of information used to present the characters are shown in **Table 4**. Movie information alone (i.e., without the addition of physical descriptors) was more often observed for movies that the participant had seen, and there was also an effect of the addressee’s knowledge such that movie information was more often used alone to present likely known than likely unknown movie characters.

**Table 4:** Effect of the participant’s personal knowledge (seen or not seen) and of the addressee’s likely knowledge (likely known or likely unknown) on the combinations of information types.

Proportions of characters presented with:			Paired *t*-test^1^	Correlation ToM^2^	Correlation IRI-PT^2^
	**Seen**	**Not seen**			
Movie information only	0.48	0.17	*t*(30) = 4.01, *p* < 0.001	*r* = −0.10, N.S.	*r* = 0.15, N.S.
Movie information and description	0.41	0.23	*t*(30) = 2.11, *p* < 0.046	*r* = −0.02, N.S.	*r* = −0.25, N.S.
Description only	0.09	0.49	*t*(30) = 5.35, *p* < 0.001	*r* = 0.08, N.S.	*r* = 0.05, N.S.
	**Likely known**	**Likely unknown**			
Movie information only	0.62	0.44	*t*(34) = 2.84, *p* < 0.007	*r* = 0.16, N.S.	*r* = 0.19, N.S.
Movie information and description	0.27	0.42	*t*(34) = 2.41, *p* < 0.022	*r* = −0.26, N.S.	*r* = 0.02, N.S.
Description only	0.08	0.12	*t*(34) = 0.85, N.S.	*r* = 0.19, N.S.	*r* = −0.29, N.S.

*ToM, Theory of Mind. IRI-PT, the empathic perspective-taking scale from the Interpersonal Reactivity Index (Davis, 1980).*

*^1^Participants who did not have data in both categories for a given comparison were excluded from the analyses. ^2^A positive correlation means that participants with a higher score on the social cognition measures used relatively more of the targeted information type for the Seen or for the Likely known conditions, while a negative correlation reflects that they used relatively more of that information type for the Not seen or the Likely unknown condition.*

Interestingly, participants also more often used movie information accompanied by description when they had seen the movie than when they had not seen it, but this time the opposite pattern was observed when considering the addressee’s likely knowledge. Such combinations of movie information and description were indeed more often employed when the addressee was unlikely to know the character.

On the other hand, the use of description only (i.e., without movie information) was significantly influenced only by the participants’ own knowledge and not by the addressee’s likely knowledge.

No significant correlation was observed between adjustment in these measures and performance on the social cognition tests. Given our a priori hypotheses, the correlation results are nonetheless included in **Table 4** for ToM and Perspective taking.

## Discussion

This study examined the effect of speakers’ personal knowledge and of their addressee’s likely knowledge on the referential expressions produced during a collaborative referential communication task. The task asked participants to verbally present movie characters to their addressee who had to identify these characters and place them in order. During the task, participants’ referential expressions were influenced not only by their own knowledge but also by their addressee’s likely knowledge of the characters, even if their addressee’s knowledge of the characters had not been previously established and was thus only probabilistic. Interestingly, a significant relationship emerged between performance on a ToM test and the increased amount of information given for the likely unknown characters. These results suggest that speakers use ToM to infer their addressee’s likely knowledge and accordingly adapt their referential expressions during verbal interactions.

### Referential Choices and Addressee’s Likely Knowledge

When presenting characters that were likely unknown from their addressee, participants spontaneously used a greater amount of information and in particular increased their use of physical descriptors. Interestingly, these physical descriptors were most often employed in combination with movie-related information. These results replicate Heller et al.’s (2012) findings in showing that speakers are not strictly Gricean in their choices of referring expressions, often choosing to over specify their expressions by including information that was not previously in common ground with their addressee. Just as participants used shape names that their addressee had not learned in the study by Heller et al. (2012) here the participants used movie related information even when the addressee was unlikely to know the movie character. In fact, when the participant knew the characters, they infrequently used only physical description, doing so for only 8% of the likely known trials and 12% of the likely unknown trials, with no significant difference between these two conditions. Despite these low amounts of trials with only descriptive information, our data provide evidence that speakers took their addressee’s likely knowledge of the characters into account. When presenting characters that were likely not known by their addressee, participants spontaneously produced more instances of movie information accompanied by physical description and fewer instances of movie information without physical description, this adjustment occurring in advance of the clarification demands from the addressee. Overall, these results suggest that the addressee’s likely knowledge was indeed taken into account by speakers for the production of their referential expressions even if the addressee’s knowledge has not been previously established and thus remained probabilistic. This observation is in line with Brown-Schmidt’s (2012) suggestion of a gradient representation of common ground that goes beyond the “*is in common ground*” or “*is in privileged ground*” dichotomy.

While the participants were sensitive to their addressee’s likely knowledge, there was always a chance that the addressee would know the characters, even for the ones who are less well known. It is thus possible that acknowledgment of that possibility by our participants made them use more movie related information. This idea would also be consistent with the observation that the proportion of trials presented using only description was lower in this study relative to the previous study by Heller et al. (2012) where some shapes were certain not to be known from the addressee, being abstract shapes introduced to the speaker specifically for the experimental task. Another possibility is that the participants in this study may have wanted to display their movie-related knowledge to their partner during the social interaction. While providing too little information in the context of this type of task may be seen as reflecting lesser collaborative tendencies, providing more information does not prevent the addressee from identifying the character and could even promote social interactions in a real-life context.

It is also quite interesting that the type of movie-related information being used in our study differed depending on the likely known or likely unknown nature of the stimuli, a result that we had not initially expected. The results revealed that while names were, as predicted, more often used for likely known characters, movie titles were in contrast used more often for likely unknown characters. Because this effect disappeared when considering only the first information provided for each trial, it suggests that movie titles were added after another initial piece of information, for example saying “*it is Wolverine from the movie X-man.*” It is thus possible that movie titles were thought to have a greater chance of being known by the addressee than the character’s names for these likely unknown trials. In real life, people often hear about movies that they have not necessarily seen, and having even a vague recollection of what the movie is about could potentially help the addressee to distinguish between the different characters. We cannot, however, exclude that the participants could have had more difficulty recollecting the names of the characters that are less well known, a possibility that would have to be controlled for in future studies. However, regardless of whether the type of movie related information used was linked to the addressee’s likely knowledge or to the participant’s own ability to recollect the information about the characters, it remains that participants adapted their references to their addressee by more often including relevant physical descriptors to which their addressee had visual access when presenting likely unknown characters. This observation clearly suggests an adjustment for the addressee even when the other person’s knowledge is probabilistic and varies for different items that the speaker and addressee encounter together for the first time.

Isaacs and Clark (1987) had previously shown that speakers adapt the content of their referential expressions based on the knowledge that their addressee held prior to any interaction around the task material. In their study, however, they did not distinguish between the likely knowledge of different items. What differed was instead the addressee’s general knowledge of the whole set of stimulus images, in that case New York scenes. The adjustment thus did not have to be readjusted on an item-by-item basis. Here, we showed that participants can continuously adapt their verbal productions depending on the likely knowledge linked to each individual image in a card set. The adjustment that we observed thus definitely goes beyond the use of a simple information overlap heuristic where the speaker would be influenced by how much knowledge he shares overall with his addressee, as was previously suggested by Wu and Keysar (2007).

The timing of the adjustment would, however, require further investigation as it remains unclear if the adjustments observed in this study were initially prepared or rather occurred through monitoring and adjustment mechanisms. The exploratory analyses on the first pieces of information provided for each trial showed a trend-level effect of the addressee’s likely knowledge, with more descriptions and less characters’ names as first pieces of information for likely unknown movies. These effects were, however, more pronounced and significant when considering all the information provided by the speaker, making it hard to distinguish if these additional information were initially planned or added later in the verbal production process. While this question would require further investigation, a recent magneto-encephalography study has provided evidence that some brain regions involved in collaborative interactions get activated even before the collaborative task trials, when subjects receives evidence that common ground knowledge necessary for the task is going to be needed shortly (Stolk et al., 2013).

### Referential Choices and Theory of Mind (ToM)

An important finding of this study is that better ToM performance was related to a greater adjustment to the addressee’s likely knowledge. These results thus support the idea that speakers can use their ToM abilities to infer their addressee’s likely knowledge and accordingly adapt their referential expressions. Even if it is widely recognized that speakers use information about what is in common ground with their addressee during verbal interactions, very few studies have directly tested the involvement of the cognition processes allowing a proper evaluation of other people’s knowledge, i.e., social cognition processes. Among the different social cognition processes, ToM is thought to be more closely related to everyday interactions because it allows mental state inferences while flexibly taking into account all available information about the person and about the context in which that person is placed. It is also more closely linked to everyday functioning in clinical populations such as patients with schizophrenia (Fett et al., 2011; Achim et al., 2012, 2013b), suggesting that this ability is important for everyday interactions. In this study, the addressee’s likely knowledge had to be inferred for each item of the experimental task, as it was not formerly established before the task as in Heller et al. (2012) and it was not constant across task items as in Isaacs and Clark (1987). Given that our participants had to continuously assess their addressee’s knowledge for each item during the task, it is understandable that the ToM measure was the one that showed a significant correlation with the adjustment of the referential expressions to the addressee’s inferred knowledge in the current task. Though we were also expecting a link with the empathic perspective-taking questionnaire, this relationship was in the expected direction but did not reach significance. It remains unclear whether this lack of a significant relationship with perspective-taking was linked to the nature of our measure (i.e., a self-report questionnaire) or to the lack of statistical power to detect more subtle effects. Studies with greater sample sizes and greater number of trials would definitely be required to answer that question.

### Limitations

The limitations of this study include that the number of stimulus items was limited, which required using random permutations to provide a proper estimate of the probability for our *t*-values. This study provides initial evidence for an adjustment to an addressee’s likely knowledge, but future studies should definitely reproduce these results using a greater number of items. Also, our group of participants was relatively small, which may have limited in particular the ability to detect significant correlations.

Another important limitation of this study is that we used the information regarding whether participants had seen the movie or not as a proxy for knowing the characters (i.e., we distinguished characters from movies that the participants had seen or not seen). As such, we did not have access to information regarding the knowledge that our participants had of the names, movie titles and roles associated to each character in our stimulus set. It is thus likely that our participants in fact knew some of the characters from the movies they had not seen, an idea supported by the observation that participants sometimes used movie-related information for characters from movie that they had not seen. It is also likely that some participant knew only part of the movie related information for some characters from movies they had seen, for example not remembering the character’s name but remembering his role and the movie title. Furthermore, it is also possible that our participants sometimes incorrectly estimated their addressee’s likely knowledge. These factors could certainly be taken into account in future studies, which could lead to the detection of stronger effects.

This study could have also been limited by the use of a confederate who acted as the addressee (Kuhlen and Brennan, 2013). The decision to use a confederate was taken because we wished to control for the knowledge displayed by the addressee and the feedback given to the different participants, reflecting knowledge of all likely known movie characters, and absence of knowledge for all likely unknown characters. In fact, the addressee was familiar with all the characters before the task, which may have influenced their pattern of feedback (Kuhlen and Brennan, 2013). However, the influence of their actual knowledge could have only suggested an increased knowledge for the likely unknown characters, hence reducing the difference between likely known and likely unknown characters. The fact that significant effects were nonetheless observed leads us to believe that this strategy did not have a detrimental effect in the present study.

In addition, it remains possible that the timing of the addressee’s feedback could have influenced the results. The addressees received no specific instructions regarding the timing of their feedback and it is possible that even short silences could have been taken by the speakers as reflecting confusion and hence a need for further information, possibly prompting the addition of additional information by the speaker.

Another limitation of this study is that it included too few women to reliably address the question of possible differences in choices of referential expressions between men and women. Exploration of the results separately in men and women suggested similar patterns in both genders for the amount of information provided per character and for the combinations of information used to present the characters, although the effects did not typically reach significance in the small group of women. While some of the effects observed in the whole group for the different types of movie-related information did not seem to emerge in women (i.e., the effects for names and for movie-titles), it is unclear if eventual gender differences would be linked to the participants’ gender per see, or to the very limited number of likely unknown movies that most of these women actually knew. This question would definitely deserve further investigation in a study including a greater number of women with different patterns of personal knowledge of movie characters.

## Conclusion

Despite the reported limitations, a significant and interesting pattern of results emerged showing that our participants were sensitive to their addressee’s likely knowledge. These results add to the field by showing that speakers adapt their verbal productions to their addressee even when common ground is probabilistic, a result that we observed while taking the participant’s own knowledge into account. In addition, the results of the current study suggest that this adjustment to the addressee takes the form of an increased amount of information provided when the addressee may not know the referent, which was done in advance of the clarification demands from the addressee and was linked to participant’s ToM abilities. This relationship with ToM abilities is an important observation, as such a link with verbal production had been previously proposed (Brennan et al., 2010) but not formerly demonstrated in healthy participants.

## Conflict of Interest Statement

The authors declare that the research was conducted in the absence of any commercial or financial relationships that could be construed as a potential conflict of interest.
